# Glucosylsphingosine Is a Highly Sensitive and Specific Biomarker for Primary Diagnostic and Follow-Up Monitoring in Gaucher Disease in a Non-Jewish, Caucasian Cohort of Gaucher Disease Patients

**DOI:** 10.1371/journal.pone.0079732

**Published:** 2013-11-20

**Authors:** Arndt Rolfs, Anne-Katrin Giese, Ulrike Grittner, Daniel Mascher, Deborah Elstein, Ari Zimran, Tobias Böttcher, Jan Lukas, Rayk Hübner, Uta Gölnitz, Anja Röhle, Ales Dudesek, Wolfgang Meyer, Matthias Wittstock, Hermann Mascher

**Affiliations:** 1 Albrecht-Kossel-Institute for Neuroregeneration, University of Rostock, Rostock, Germany; 2 Department for Biostatistics and Clinical Epidemiology, Charité-University Medical Centre, Berlin, Germany; 3 pharm-analyt Labor GmbH, Baden, Austria; 4 Gaucher Clinic, Shaare Zedek Medical Center, Jerusalem, Israel; 5 Centogene GmbH, Rostock, Germany; 6 Department of Neurology, University of Rostock, Rostock, Germany; 7 Queen Mary University of London, Barts and the London School of Medicine and Dentistry, London, United Kingdom; University Hospital S. Maria della Misericordia, Udine, Italy

## Abstract

**Background:**

Gaucher disease (GD) is the most common lysosomal storage disorder (LSD). Based on a deficient β-glucocerebrosidase it leads to an accumulation of glucosylceramide. Standard diagnostic procedures include measurement of enzyme activity, genetic testing as well as analysis of chitotriosidase and CCL18/PARC as biomarkers. Even though chitotriosidase is the most well-established biomarker in GD, it is not specific for GD. Furthermore, it may be false negative in a significant percentage of GD patients due to mutation. Additionally, chitotriosidase reflects the changes in the course of the disease belatedly. This further enhances the need for a reliable biomarker, especially for the monitoring of the disease and the impact of potential treatments.

**Methodology:**

Here, we evaluated the sensitivity and specificity of the previously reported biomarker Glucosylsphingosine with regard to different control groups (healthy control vs. GD carriers vs. other LSDs).

**Findings:**

Only GD patients displayed elevated levels of Glucosylsphingosine higher than 12 ng/ml whereas the comparison controls groups revealed concentrations below the pathological cut-off, verifying the specificity of Glucosylsphingosine as a biomarker for GD. In addition, we evaluated the biomarker before and during enzyme replacement therapy (ERT) in 19 patients, demonstrating a decrease in Glucosylsphingosine over time with the most pronounced reduction within the first 6 months of ERT. Furthermore, our data reveals a correlation between the medical consequence of specific mutations and Glucosylsphingosine.

**Interpretation:**

In summary, Glucosylsphingosine is a very promising, reliable and specific biomarker for GD.

## Introduction

Gaucher disease (GD) is the most common lysosomal storage disorder (LSD), with an estimated prevalence in Caucasians ranging from about 1∶40,000 to 1∶60,000, although a current newborn screening in Szeged, Hungary suggests a higher prevalence of 1∶13,341 [1]–[2]. LSDs are generally characterized by a genetic defect in proteins and enzymes involved in the lysosomal degradation of macromolecules into smaller compounds resulting in the accumulation of non-degraded macromolecules [3]. In GD this results in the accumulation of glucosylceramides as the enzyme β-glucocerebrosidase is impaired. This causes a compensatory lipid re-uptake by macrophages which likewise cannot degrade glucosylceramides and thus enlarge and evolve into the disease-specific “Gaucher cells”. They are the hallmark of the disease [4].

Depending on onset and symptoms, GD can be classified as non-neuronopathic, which is the mildest and most common phenotype in GD; the acute, neuronopathic form, which represents the severest form of the disease and is fatal within a few years; and the sub-acute, chronic neuronopathic form [5]–[6]. Non-neuronopathic GD is the most common disease manifestation in the Ashkenazi Jewish population and with a frequency of up to 1∶1,000, it is far more common than in the European Caucasian population [7]. Genotype–phenotype correlations have been described in the literature. The most common examples are the mutation N370S usually detected in patients with non-neuronopathic GD carrying a milder burden of disease whereas e.g. the mutation L444P is more frequently seen in the more severe neuronopathic form of GD [1].

Established treatment options entail either enzyme replacement therapy (ERT) with recombinant glucocerebrosidase (Cerezyme™, Genzyme Corporation, Cambridge, MA USA; or VPRIV™, Shire HGT, Lexington, MA USA) that supplement the missing or malfunctioning enzyme or substrate reduction therapy (SRT) with Miglustat (Zavesca™, Actelion Pharmaceuticals Ltd, Allschwil, Switzerland) which reduces production of the available substrate.

Current diagnostic steps comprise the measurement of β-glucocerebrosidase enzyme activity in fibroblasts and leucocytes, supported by the detection of the mutation and determination of chitotriosidase and CCL18/PARC [8]. However, enzyme activity levels cannot be used to reliably determine disease severity. The routinely available biomarkers, chitotriosidase and CCL-18, are epiphenomena caused by activation of macrophages after uptake of glucosylceramide. Therefore, they do not reflect the pathophysiology of the disease and reflect only to a limited extent the disease activity or response to therapy. While GD patients display a massive increase in chitotriosidase, patients with other LSDs exhibit elevated chitotriosidase levels to a lesser extent. However this limits the significance and value of this measurement [9]. In male Fabry patients Vedder and colleagues found evidence of elevated chitotriosidase levels which was reduced to normal after onset of treatment reflecting the lipid accumulation in Fabry patients in macrophages prior to therapy [10]. Not only lysosomal storage disorders may cause an increase in chitotriosidase, peroxisomal disorders like the X-linked cerebral adrenoleukodystrophy may also cause an elevation of this biomarker, which recently was reported to be able to monitor and predict the prognosis of patients with X-linked cerebral adrenoleukodystrophy receiving allogeneic hematopoietic stem cell transplantation [11].

Furthermore, subjects, including those with GD, may have a chitotriosidase activity deficiency due to a 24–base pair (bp) duplication in the chitotriosidase gene. Obviously, these individuals cannot be monitored by the measurement of plasma chitotriosidase activity [12]–[13]. The frequency of the homozygous 24-bp duplication in the chitotriosidase gene depends on the ethnicity and can vary between 6% and nearly 35% in the Latino population (unpublished data). In these cases the marker CCL18 is used [14]–[15]. However, elevated levels of CCL18 were also found to be associated with a variety of diseases, such as different types of cancer and inflammation of joints, lungs and skin (e.g. rheumatoid arthritis, hypersensitivity pneumonitis and atopic dermatitis, for details see review by Schutyser and colleagues) [16]. For example, the ascitic fluid of patients suffering from ovarian cancer contains a significantly elevated level of CCL18 compared to patients without this carcinoma (Budd-Chiari syndrome) [17]. Since it attracts and activates specific immune cells CCL18 plays a role in tumor suppression. Furthermore, children having acute lymphocytic leukemia are found to exhibit elevated levels of CCL18 [18]. Hence, plasma levels of CCL18 do not reflect one particular clinical symptom, but are rather a reflection of the total body burden of Gaucher cells.

Therefore, there is a definite need for a sensitive and specific biomarker, which would need to be feasible for diagnosis of GD as well as follow-up monitoring of GD patients.

Biomarkers should ideally reflect or be involved in the pathophysiology of the given disease. They should change in parallel with the burden of the disease and effectively discriminate between affected and non-affected probands. Some parameters being involved in the pathologically changed metabolic pathway had been analysed in the past: Groener et al. have analysed the ratio of Glucosylceramide/Ceramide (GlcCer/Cer) to discriminate between GD patients and healthy patients [19]. GlcCer and Cer were measured with high performance liquid chromatography (HPLC) essentially as described by Groener and colleagues [20]. The level of GlcCer was found to be different although ceramide levels were not significantly different in the plasma of treated and untreated GD type I patients.

Recently, Dekker and colleagues introduced glucosylsphingosine, which was first reported to be elevated in the cerebrum and cerebellum of patients with acute and subacute neuronopathic GD [21], as a new marker in the blood plasma of GD patients [22]. The authors demonstrated that plasma glucosylsphingosine levels were correlated well with chitotriosidase and CCL18. Furthermore, treatment of GD resulted in a clear glucosylsphingosine reduction, similar to chitotriosidase. Currently, there are also approaches to identify feasible markers for bone turnover in GD [23].

In order to establish a sensitive and specific biomarker for GD, we compared mass spectra of the plasma of healthy controls and GD patients using HPLC and tandem mass spectrometry. Mass spectra that differed most between patients and controls were analysed in more detail. The resulting biomarker, which was patented in June 2011 (PCT/EP2012/002409; Arndt Rolfs, Hermann Mascher), was Glucosylsphingosine. We identified this compound independent of Dekker and colleagues as a reliable, sensitive and specific biomarker for GD in a large cohort of GD patients. Furthermore, we evaluated whether Glucosylsphingosine is related to the specific genotypes and is reliable for long-term monitoring of the efficiency of therapy.

## Results

Overall, 551 non-Jewish, Caucasian subjects were analysed. The population consisted of 272 (49.6%) males and 272 (49.6%) females, in 7 cases the gender was not known. In total 148 healthy controls were compared to 98 genetically diagnosed GD patients (for detailed information see Table S1), 13 GD carriers, and 262 patients with other LSDs (Table 1). Patients with other LSDs were suffering from Niemann-Pick-Type C disease, Krabbe disease, Hunter disease and Fabry disease. There were significant differences in the gender distribution when comparing the four sub-cohorts (p = 0.029) as more males were in the GD carrier group than in the other three sub-cohorts. There was a significant age difference as the healthy control group and the GD patient group were younger than the GD carriers and patients with other LSDs (p = 0.012). The healthy controls, 81 (54.7%) males and 67 (44.8%) females, had a median age of 29.0 years, while the GD carriers, 8 (64.7%) males and 5 (35.3%) females, had a median age of 35.0 years. Of the GD patients 66 (52.26%) were males and 56 (47.8%) were females and had overall a median age of 29.0 years. The patients suffering from other LSDs had a median age of 38.0 years and consisted of 117 (44.8%) males and 144 (55.2%) females.

**Table 1:** Comparison of the four cohorts.

	Healthy Controls			GD Carriers		GD Patients		Other LSDs	
**N individuals**	148			13		129		261	
**%**	26.9			2.4		23.4		47.4	
**N measures**	163			15		456		340	
**Age in years:**									
**Median**	29			35		29		38	
**Interquartile Range**	(5–48)			(31–59)		(8–44)		(19–50)	
**Number of Cases**	(n = 141)			(n = 13)		(n = 119)		(n = 238)	
	male		female	male	female	male	female	male	female
**n**	81	67		8	5	66	56	117	144
**%**	54.7	44.8		64.7	35.3	52.2	47.8	44.8	55.2
**Age in years:**									
**Median**	26	34		34	39	24	32	31	44
**Interquartile Range**	(5–50)	(5–47)		(26–52)	(33–70)	(8–45)	(10–43)	(14–49)	(25–53)

Healthy controls, GD carriers, GD patients and patients with other LSDs with respect to age and gender. 148 controls and 261 patients suffering from other LSDs were compared to 13 GD carriers and 129 patients suffering from GD.

**Figure 1:**
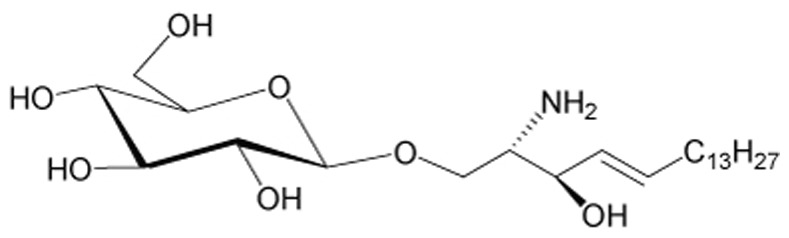
Chemical structure of Glucosylsphingosine. This substance was only analysed by HPLC-MS/MS with high sensitivity and specificity of diagnosing GD.

**Figure 2:**
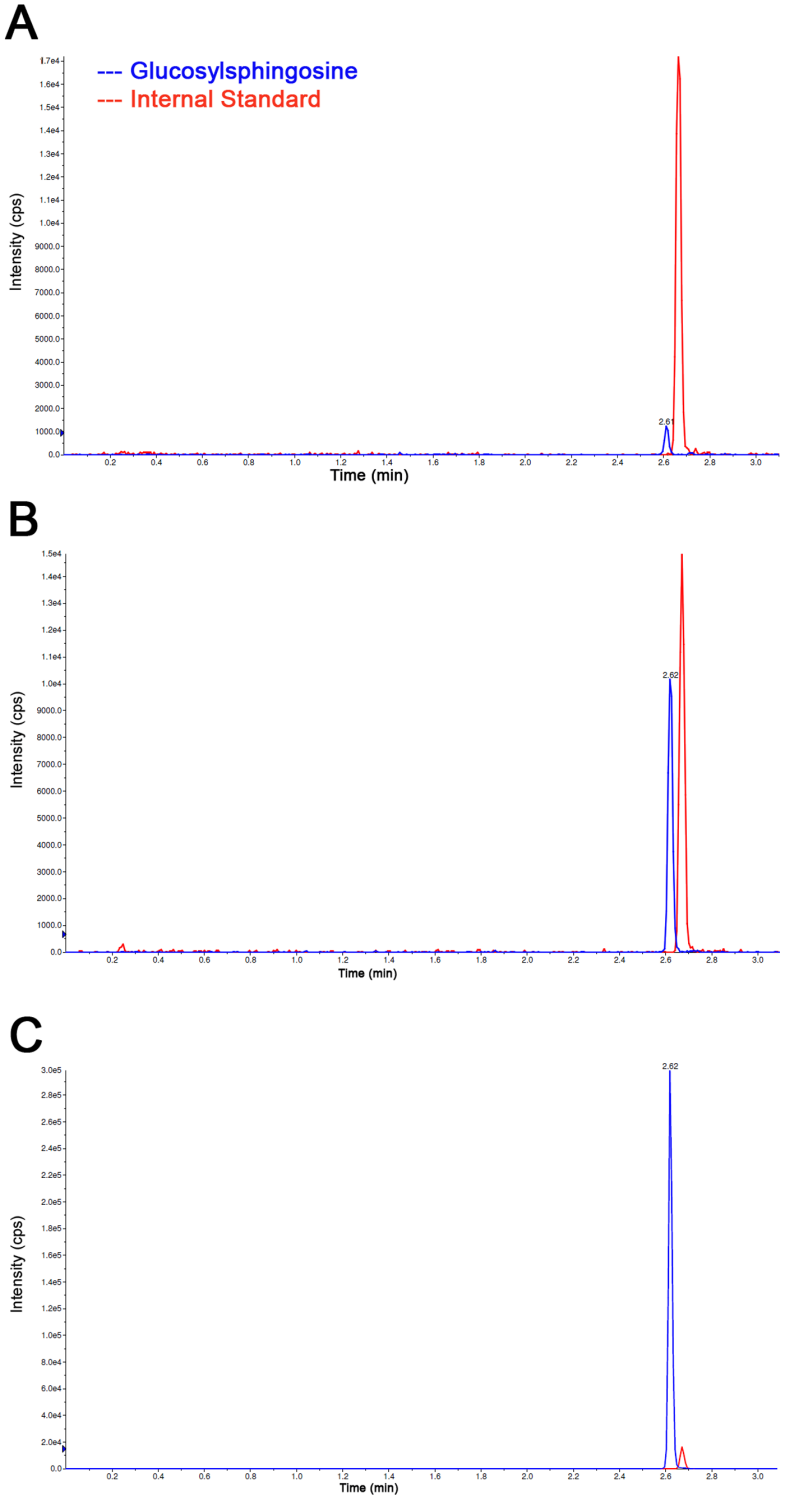
Analysis of Glucosylsphingosine and Internal Standard in different samples. Measurement of Glucosylsphingosine (first peak in chromatograms (blue), second peak in chromatograms is Internal Standard (red)) in a healthy control (1.71 ng/ml) (A) and two differently affected individuals with GD (B and C). Medium level of Glucosylsphingosine is shown for the first patient (B: 17.1 ng/ml) and a high level of Glucosylsphingosine for the second (C: 319 ng/ml). Please note the changed scale in (C).

According to the established protocol Glucosylsphingosine (chemical structure depicted in Figure 1) measurements were carried out in blood plasma samples. Exemplary measurements are depicted in Figure 2, comparing Glucosylsphingosine measurements of a healthy control (Figure 2A, blue line, first peak in chromatogram), a mildly affected GD patient (Figure 2B), and a severely affected GD patient (Figure 2C) compared to the internal standard (Figure 2, red line, second peak in chromatogram).

**Figure 3:**
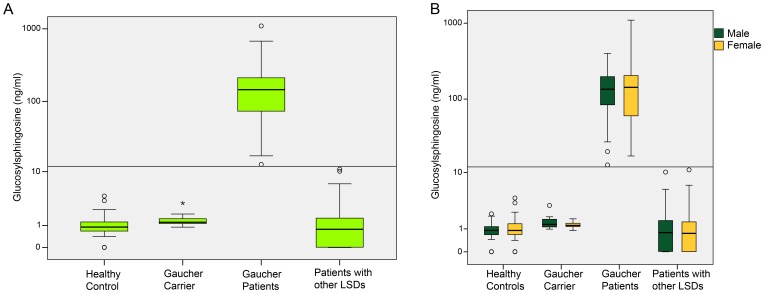
Glucosylsphingosine levels in sub-cohorts. Level of Glucosylsphingosine is illustrated in the entire cohort (A) and separated according to gender (B). Glucosylsphingosine in GD patients was compared to healthy controls, GD carriers and patients with other LSDs. The horizontal bar marks the cut-off for pathological Glucosylsphingosine values above 12 ng/ml. Notably, only GD patients feature pathological values of Glucosylsphingosine and additionally Glucosylsphingosine is not gender-dependent (B; dark green representing males and orange females).

Subsequently, Glucosylsphingosine values for all four sub-cohorts were statistically analysed. Several cut points were considered and a careful analysis yielded a cut-off of 12 ng/ml with an ideal sensitivity and specificity of 100% (data for other calculated cut-offs not shown). This observation was independent of gender (Figure 3B), levels of Glucosylsphingosine being similar in male and female GD patients. Notably, neither healthy probands, patients with other LSDs nor GD carriers exhibited pathologic values above the cut-off of 12 ng/ml (Figure 3A). Only GD patients displayed Glucosylsphingosine values above the normal range.

Both Chitotriosidase and CCL18 were quantified as well, and sensitivity and specificity were compared to those of Glucosylsphingosine (Table 2). Chitotriosidase had a sensitivity of 91.7% and a specificity of 86.1%. In our analysed patients and controls CCL18 had a sensitivity of 76.2% and a specificity of 79.4%.

**Table 2:** Sensitivity and specificity for different biomarkers with regard to diagnosis of GD.

	Chitotriosidase (n = 233)	CCL18 Park (n = 207)	Glucosylsphingosine (n = 521)
Cut point	>145	>166	>12
Sensitivity	91.7%	76.2%	100.0%
Specificity	86.1%	79.4%	100.0%
AUC and 95%CI in ROC Analysis	0.94 (0.89–0.98)	0.87 (0.80–0.93)	1.00 (1.00–1.00)

So far Chitotriosidase is the standard biomarker for GD, however due to mutations in the Chitotriosidase gene, Chitotriosidase can be false negative, thus makes the use of the far more unreliable marker CCL18 necessary. For assessment of chitotriosidase sensitivity and specificity 233 samples were analyses resulting in a sensitivity of 91.7% and a specificity of 86.1%. Overall 207 samples were analysed to evaluate the sensitivity and specificity of CCL18, which displayed a sensitivity of 76.2% and a specificity of 79.4% each. We propose Glucosylsphingosine as a more specific and sensitive biomarker for GD as it yielded both a sensitivity and specificity of 100% in 521 analysed samples.

**Figure 4:**
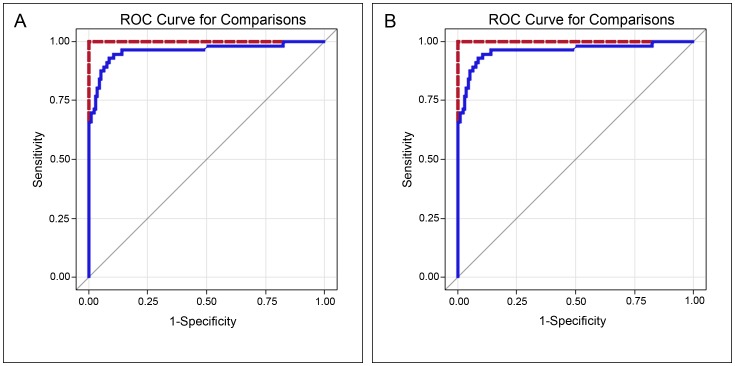
ROC curve analysis for comparison of Glucosylsphingosine. Glucosylsphingosine (A; red line; area under the curve (AUC) = 1.00) and Chitotriosidase (A; blue line; AUC = 0.96) as well as Glucosylsphingosine (B; red line; AUC = 1.00) and CCL18 (B; blue line; AUC = 0.86) to discriminate the accuracy of two values. Glucosylsphingosine is significantly more accurate than Chitotriosidase (A: p = 0.027, n = 228) and CCL18 (B: p<0.001, n = 207).

Receiver Operating Characteristics (ROC) analysis was carried out to evaluate the accuracy of Glucosylsphingosine compared to chitotriosidase (Figure 4A) and CCL18 (Figure 4B). The corresponding area under the curve (AUC) and confidence intervals (CI) are 1.00 (1.00–1.00) for Glucosylsphingosine, 0.96 (0.89–0.98) for chitotriosidase and 0.86 (0.80–0.93) for CCL18 (Table 2). The accuracy of Glucosylsphingosine is significantly better than chitotriosidase (p = 0.027) and CCL18 (p<0.001).

Aside from the measurements of Glucosylsphingosine, chitotriosidase and CCL18 also GD genotyping was performed. A broad spectrum of different mutations was detected for the 98 GD patients where DNA was available, 45 different mutations in total. N370S (32.5%), L444P (22.1%) and RecNciI (8.0%) comprising about 2/3 of all mutations.

We found correlations between the two most frequent mutations N370S and L444P on the one hand and levels of the biomarker Glucosylsphingosine on the other hand (Table 3). Patients affected by the mutation L444P demonstrated higher blood plasma levels of Glucosylsphingosine (median 184.5 ng/ml) than those affected by the milder N370S mutation (median 143.1 ng/ml). Furthermore, Glucosylsphingosine was higher in homozygous (median of 143.1 ng/ml in N370S/N370S and 184.5 ng/ml for L444P/L444P) than in compound heterozygous (median of 77.1 ng/ml in N370S compound heterozygous and 107.0 ng/ml in L444P compound heterozygous) GD mutations.

**Table 3:** Glucosylsphingosine levels in patients carrying frequent Gaucher mutations.

	Homozygotes	Compound Heterozygotes
**N370S**		
N individuals (%)	8 (6.2%)	70 (54.3%)
Median (IQR) Glucosylsphingosine	143.1	77.1
	(96.6–236.3)	(33.1–164.0)
**L444P**		
N individuals (%)	16 (12.4%)	31 (24.0%)
Median (IQR) Glucosylsphingosine	184.5	107.0
	(103.0–275.3)	(70.5–246.0)

Comparison of the frequency and Glucosylsphingosine values (median and interquartile range (IQR)) in the mutation N370S predisposing for a benign phenotype and the mutation L444P highly likely to cause severe GD. Notably, homozygous mutations are associated with higher Glucosylsphingosine values in both mutations. In the mutation L444P, which is inherent in many severely affected GD patients, Glucosylsphingosine is higher than in patients with the mutation N370S. This indicates the correlation of Glucosylsphingosine with the burden of the disease as homozygous individuals are more severely affected than compound heterozygous ones and patients with L444P more than those with N370S. Of note, patients compound heterozygous for N370S/L444P were included in both compound heterozygous cohorts.

**Figure 5:**
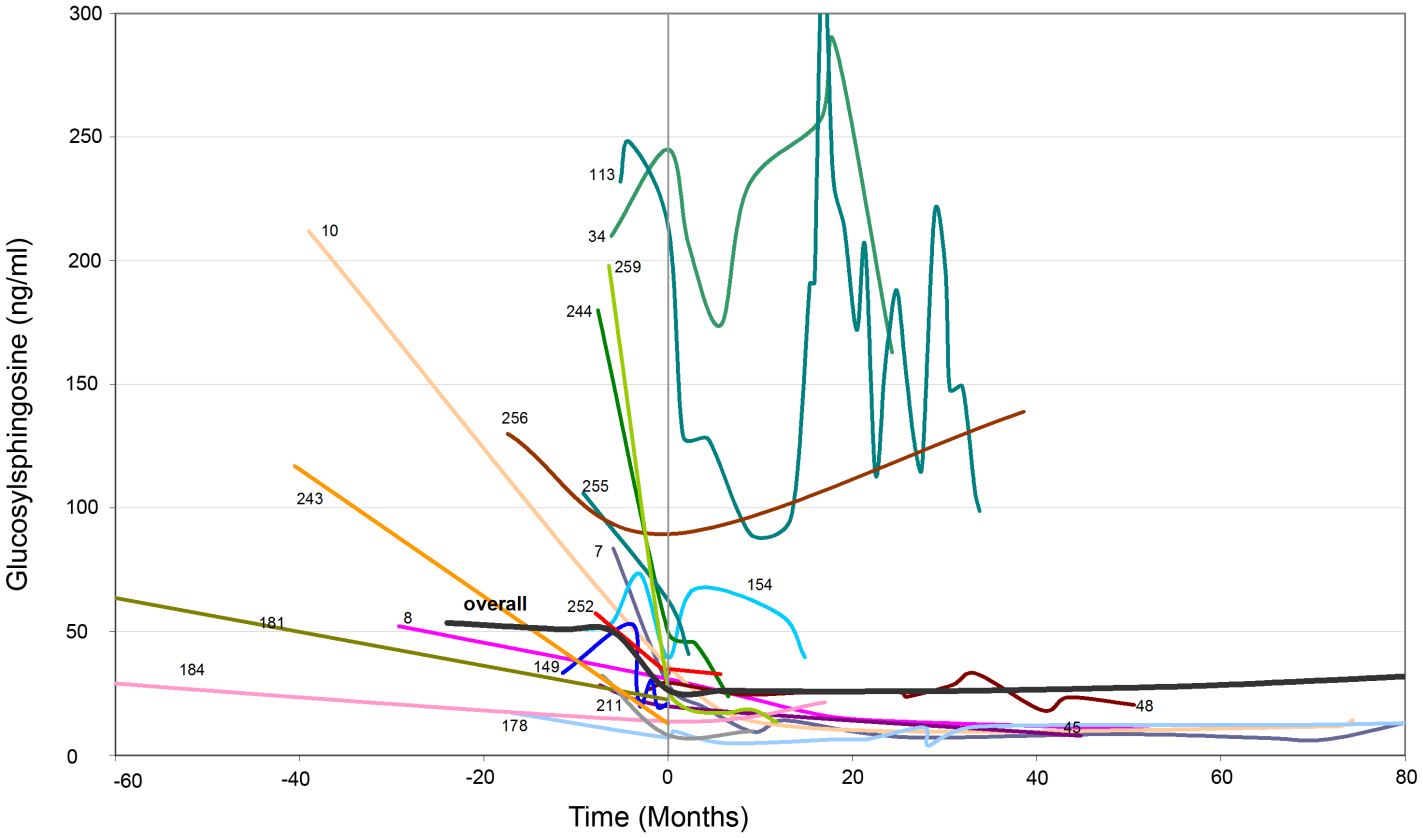
Monitoring of enzyme replacement therapy by Glucosylsphingosine. Course of Glucosylsphingosine after onset of treatment, the time point zero representing the first value after onset of therapy. The course for 19 GD patients undergoing ERT is shown. In summary the following genotypes were detected: #256: N370S/N370S; #7, #8, #181, #242: N370S/L444P; #10, #113, #149, #154, #178, #184, #211 are N370S/other. Two severely affected patients, 113 and 34, had been started with ERT for 10 months (pts. 113) and 4 months (pts. 34), respectively, but stopped due to shortage of the treatment. With the beginning of the ERT Glucosylsphingosine dropped down immediately, however went back to the original level after stopping of the ERT. After a second start with ERT 19 and 20 months, respectively, after initial starting the ERT the Glucosylsphingosine concentration dropped down again.

For 19 GD patients we were able to monitor Glucosylsphingosine before and after start of ERT (Figure 5), blood was drawn before starting the infusion. Testing of 3 patients revealed that the biomarker in plasma was stable at 4°C up to at least 22 days and hemolytic samples of EDTA-blood yielded identical results compared to those samples immediately processed after sampling. For two patients we collected plasma at several time points (24 h, 12 h, 6 h before ERT, right before the start of ERT infusion, 6 h, 12 h, 24 after ERT). The samples had a range of ±8% within the first 24 hours of ERT, indicating that there was no immediate effect of ERT on the Glucosylsphingosine levels.

Overall, the patients ranged from 50–250 ng/ml prior to ERT. Time point zero by definition characterises the first measurement after start of therapy, which not always represented the first day of ERT but also weeks after the initial treatment. This is due to the fact that this part of the study was undertaken in retrospective and blood samples at onset of therapy was not available for all patients. Thus, Glucosylsphingosine was already reduced at time point zero instead of decreasing after commencement of therapy.

For all patients a significant reduction after start of therapy and over time was detected (Figure 5A), e.g., the very striking graph of patient #34, whose Glucosylsphingosine values decrease after start of ERT, but increases significantly when the patient had to discontinue therapy due to ERT shortage. The graphs #10, #244 and #259 display a clear reduction of Glucosylsphingosine from an average 200 ng/ml before onset of therapy to levels below 50 ng/ml after onset of therapy. Overall, the vast majority of patients feature Glucosylsphingosine values below 50 ng/ml after onset of ERT (Figure 5).

A linear mixed-model regression analysis for Glucosylsphingosine before and after therapy was carried out (Table 4). Only patients with Glucosylsphingosine measurements before and after onset of therapy were included in the analysis. On average patients started with a concentration of 50.0 ng/ml Glucosylsphingosine before treatment. The most striking reduction of Glucosylsphingosine occurs right after the start of therapy (47.3%, p<0.001) and within the first 6 months (47.3%), although reduction over time is also highly significant (p<0.0001).

**Table 4:** Regression analysis for Glucosylsphingosine.

Time in months	Glucosylsphingosine(ng/ml)	Reduction in % comparedto 6 months before therapy
−6	50.0	−
0	26.4	47.3
6	26.1	47.8
12	25.9	48.2
18	25.8	48.3
24	25.9	48.2
30	26.0	47.9
36	26.3	47.4
48	27.1	45.8
60	28.4	43.1

The analysis included all 19 patients for whom values before and after ERT were available, details on the course of Glucosylsphingosine values are shown in Figure 5. Time point zero represents the first value after onset of therapy. The table shows regression based (linear mixed models) predicted values for Glucosylsphingosine and reduction in % compared to 6 months before therapy. To overcome the skewed distribution of measurements Glucosylsphingosine values were logarithmised before regression analysis.

Glucosylsphingosine was reduced after onset of ERT. The largest reduction occurred within the first 6 months after starting the ERT therapy. Patients display a reduction of 47.8% after 6 months and of 43.1% after 60 months of therapy.

## Discussion

In 1989 Rosengren and colleagues published a comparative analysis of glycosphingolipid patterns, especially lyso-sulfatide, in the normal brain and in patients suffering from Metachromatic Leukodystrophy [24]. They concluded that lyso-glycosphingolipids did not contribute to disease pathology as their findings supported the idea of de-novo-synthesis of lyso-glycosphingolipids from sphingosine. Likewise Lloyd-Evan and colleagues concluded from their experiments with glycosphingolipids and calcium that they may not contribute to disease pathology, but could play a possible role in treatment [25]. However, these experiments were conducted with healthy Wistar rats, not suffering from a specific LSD. This is contrary to the older findings of Atsumi and colleagues, who argued for a pathologic role of glycosphingolipids [26]. This is also supported by more recent studies, where Glucosylsphingosine in particular was investigated and found to be responsible for neuronal cell death [27]. The presence of glucosylceramide and glucosylsphingosine in the cerebrum and cerebellum of five infantile and juvenile Gaucher patients has been described by Nilsson and Svennerholm (1981), demonstrating the highest levels of glucosylcermide and glucosylsphingosine in the severest cases [21].

Here, Glucosylsphingosine was analysed in a large, non-Jewish, Caucasian cohort, comprising healthy probands, GD patients, GD carriers and patients with other LSDs. Not only did Glucosylsphingosine distinguish between healthy probands and those with GD disease with a cut-off of 12 ng/ml, but also between GD patients and GD carriers. In addition, the marker additionally proved to be specific for GD as patients with other LSDs did not shown signs of elevated Glucosylsphingosine (Figure 3). Further, Glucosylsphingosine proved to be independent of gender, with similar levels of the biomarker in both genders. This finding is in contrast to FD, where even symptomatic females display levels of lyso-Gb3 in a range that was 10-times lower than in males and in some cases even close to normal [28]. Furthermore, Glucosylsphingosine correlated with the genotype. Patients with N370S displayed lower levels of Glucosylsphingosine than those with L444P, which is known to be more frequent in patients with a more fatal course of disease [29]. Furthermore, patients homozygous for either N370S or L444P exhibit higher values for Glucosylsphingosine than those who were compound heterozygous with only one of those mutations (Table 3). This is in line with the findings of Dekker at al. who measured Glucosylsphingosine as well and found modest increases in Glucosylsphingosine in mildly affected patients [22]. Results for Glucosylsphingosine in GD patients were comparable to the results presented here, the median value for Glucosylsphingosine in type 1 GD patients was 230.7 nM, which approximately corresponds to 92.28 ng/ml and is in line with our results. However, there are differences in both measurement techniques. The method we have chosen for the measurement of direct Glucosylsphingosine might be of importance for the discrimination capacity of this marker. It is important to understand that glucosylsphingosine present in the plasma mainly consists of a sugar moiety and a ceramide moiety. Further, the ceramide moiety consists of a sphingosine and a fatty acid. Using older technology lipids are extracted and ceramide and glucosylceramide are deacylated by alkaline hydrolysis thus forming the lyso form, i.e. Glucosylsphingosine [30]. Subsequently, the Glucosylsphingosine thus produced is labeled with a fluorescence dye by derivatization with O-phthaldialdehyde (OPA) at the primary amine group. Afterwards the derivatized sphingoid bases were separated by reverse phase HPLC and measured by a fluorescence detector. With this technology one is able to detect total Glucosylsphingosine consisting of free Glucosylsphingosine and Glucosylsphingosine, but is not able to distinguish a level of free Glucosylsphingosine from a level of Glucosylsphingosine in a sample from a subject. Importantly, each primary amine circulating in the plasma, being sufficiently lipophilic to be extracted concomitantly with Glucosylsphingosine using an organic solvent is labeled accordingly. Thus, this may disturb the detection of cleaved Glucosylsphingosine. The level of said total Glucosylsphingosine after cleavage of the various fatty acid moieties from the NH2 group of the Glucosylsphingosine is usually in a range of from 5 to 30 µg per mL plasma or serum. We were able to measure free Glucosylsphingosine in the plasma without performing a cleavage of the fatty acid moieties. Therefore, this procedure allowed reducing the detection limit to 0.25 ng/ml (95 percentile). The putative advantage of our slightly different measurement approach of Glucosylsphingosine compared to the method of Dekker and colleagues is the ability to differentiate between GD patients on the one hand and GD carriers and patients with other lysosomal storage disease on the other hand, however this will have to be verified in future studies with a larger cohort and more long-term data.

In comparison to chitotriosidase and CCL18, Glucosylsphingosine proved to be 100% sensitive and 100% specific. The major disadvantage of chitotriosidase is the prevalence of the 24 base-pair duplication within approximately 6% of the normal population, which contributes to false negative results (Table 2) [9]. Another factor that complicates use of chitotriosidase, is the fact that it is a by-product amassed in Gaucher cells and not directly disease-related as Glucosylsphingosine is [31]. Since it responds very slowly, requiring about 3–6 months after start of treatment to start to decline, it hinders accurate monitoring by chitotriosidase of therapeutic interventions. Importantly, we observed a rapid and pronounced reduction of Glucosylsphingosine directly after onset of therapy (Figure 5). The pathophysiological relevance of a reduction of Glucosylsphingosine remains to be illustrated. It is yet undetermined how the reduction of Glucosylsphingosine during treatment correlates with clinical improvement. Ongoing studies (Clinicaltrial.gov, NCT01331642) will determine if the marker will allow the calculation of individual dosages and improve the clinical outcome for each patient. Overall, this highlights the potential of Glucosylsphingosine as a reliable marker for monitoring.

In summary, Glucosylsphingosine is a very promising, reliable and specific biomarker for GD. The marker is not only valid for the primary diagnosis of GD patients but also able to reflect the progress as well as improvement of the disease, e.g. when ERT has been stopped in a patient, significant increase of the concentration of the marker in plasma can be demonstrated within 3 weeks after stopping the ERT, compared to 10 weeks in case of chitotriosidase (data not shown). However, in order to characterise the correlation with clinical phenotype, the response to ERT therapy, or even to demonstrate differences in the efficiency of the different treatment options available to the patients, Glucosylsphingosine has to be analysed in further detail.

## Methods

### Patients and Blood Samples

Blood samples were obtained from patients undergoing biochemical analysis or genetic testing for verification of a suspected metabolic disease by the Albrecht-Kossel-Institute for Neuroregeneration (AKos). All patients agreed for testing of their blood samples. The protocol of the study has been approved by the local Ethical Committee of the University Rostock. Patients undergoing therapy were treated according to standard protocols. Written informed consent was obtained from all participants.

### Biochemical and Genetic Analysis

Standard analysis of GBA gene (for specific genotypes see Table S1), CCL18/PARC and Chitotriosidase were performed according to standard protocols [32]–[33].

### Identification of Glucosylsphingosine as a Biomarker for GD

Screening for potential biomarkers was carried out using HPLC-MS/MS of blood plasma samples of 10 healthy subjects and 10 GD patients with accurate mass MS-systems (Orbitrap XL) and tandem MS systems. Differences between GD patients and healthy people were carefully assessed with regards to substances strongly increased in patients. Molecular weight and structure of such substances were determined carefully. Peaks identified by tandem MS/MS were measured using already known internal standards as reference. Successive measurement in larger GD cohorts ensued and after validating the robustness of Glucosylsphingosine, measurement in healthy cohort, GD carriers, GD patients and in patients with other LSDs followed.

After the first block of analysis of GD patients (where the information healthy – not healthy was known) all other analyses for Glucosylsphingosine were done blinded.

### Method for Determination of Free Glucosylsphingosine in Plasma

50 µL of the sample are mixed with 100 µL of Internal Standard working solution (in EtOH), are thus mixed subsequently using a DVX-2500 Multi-tube vortex device at 2500 rpm for about 30 seconds. After centrifugation at 4000 rpm for 2 minutes the clear supernatant was transferred into auto-sampler vials and injected into the HPLC-MS/MS system. Mobile phase used for gradient elution was 50 mM formic acid in water and 50 mM formic acid in acetonitrile/acetone (1/1, v/v). HPLC flow was set at 0.9 mL/min on an ACE 3 C8 column (50×2.1 mm) at 60°C, injection volume used was 5 µL. Retention time for the analyte was approximately 3.4 minutes and for the internal standard (lyso-Gb2) approximately 3.6 minutes. Lyso-Gb2 was checked in regards to native concentrations in plasma and was found to be at very low levels only, sufficient amount was added therefore during sample preparation. The MS/MS system used was an API 4000 using electrospray ionization in MRM mode in positive mode at 500°C for determination of free Glucosylsphingosine in plasma. Quadrupole resolution was set at unit/unit, MRM transitions used were 462 → 282 m/z for the analyte and 624 → 282 for the internal standard. Calibration was done from 0.4 ng/mL to 100 ng/mL in methanolic solution, QC samples were spiked in plasma at levels of 1, 5, and 50 ng/mL.

### Statistics

In order to compare the diagnostic value of the different biomarkers and for the calculation of correlations between the biomarkers, we first aggregated the data using the earliest measured value of every marker for GD patients before therapy and the highest value for non-GD for a particular patient if more than one blood sample was available. This resulted in a sample of 148 healthy controls, 13 GD carriers, 129 GD patients and 261 patients with other LSDs.

The accuracy of values of the different biomarkers (Glucosylsphingosine, chitotriosidase, enzyme activity and CCL18/PARC) to discriminate patients with GD disease from patients without GD was evaluated using Receiver Operating Characteristic (ROC) curve analysis [34]–[35].

The area under the curve (AUC) and the 95% confidence limits for the different biomarkers are reported in Table 2. Paired sample statistical techniques were used for the comparison of two biomarkers. The method exploits the mathematical equivalence of the AUC to the Mann-Whitney U-statistic [36].

The ROC curves were calculated using PASW Statistics 18, Release Version 18.0.2 (© SPSS, Inc., 2009, Chicago, IL, www.spss.com). The comparisons of ROC curves and the linear mixed models were done using SAS software, Version 9.2 of the SAS System for Windows (© 2008 SAS Institute Inc., Cary, NC, USA).

For analysing how Glucosylsphingosine changed over time in GD patients, we analysed non-aggregated data for those patients for whom we had more than one blood sample available (20 GD patients). The first measurement under therapy for every patient was set to time point zero. We used linear mixed-models for testing if time dependent reduction – values after start of therapy compared to values before therapy – were significant.

## Supporting Information

Table S1 **Genotypes for all included GD patients.** GBA cDNA accession number NP_000148.2; Traditional amino acid residue numbering, which excludes the first 39 aminoacids of the leader sequence, is provided and designed without the prefix “p”. All detected genotypes are listed and have been confirmed by Sanger sequencing. The cohort is comprised of a non-Jewish, Caucasian cohort of GD patients.
(PDF)

## References

[1] Wang RY, Bodamer OA, Watson MS, Wilcox WR (2011) ACMG Work Group on Diagnostic Confirmation of Lysosomal Storage Diseases. Lysosomal storage diseases: diagnostic confirmation and management of presymptomatic individuals. Genet Med 13: 457–84. 21502868 10.1097/GIM.0b013e318211a7e1

[2] Wittmann J, Karg E, Turi S, Legnini E, Wittmann G, et al (2012) Newborn screening for lysosomal storage disorders in Hungary. JIMD Reports 6: 117–25. 23430949 10.1007/8904_2012_130 PMC3565645

[3] Nakamura K, Hattori K, Endo F (2011) Newborn screening for lysosomal storage disorders. Am J Med Genet C Semin Med Genet 157: 63–71. 10.1002/ajmg.c.30291 21312327

[4] Aerts JM, Kallemeijn WW, Wegdam W, Joao Ferraz M, van Breemen MJ, et al (2011) Biomarkers in the diagnosis of lysosomal storage disorders: proteins, lipids, and inhibodies. J Inherit Metab Dis 34: 605–19. 21445610 10.1007/s10545-011-9308-6 PMC3109260

[5] Hollak CE, de Fost M, van Dussen L, Vom Dahl S, Aerts JM (2009) Enzyme therapy for the treatment of type 1 Gaucher disease: clinical outcomes and dose – response relationships. Expert Opin Pharmacother 10: 2641–52. 19743939 10.1517/14656560903270520

[6] Pastores GM (2010) Neuropathic Gaucher disease. Wien Med Wochenschr 160: 605–8. 21221912 10.1007/s10354-010-0850-x

[7] Aviner S, Garty BZ, Rachmel A, Baris HN, Sidransky E, et al (2009) Type 2 Gaucher disease occurs in Ashkenazi Jews but is surprisingly rare. Blood Cells Mol Dis 43: 294–7. 19734074 10.1016/j.bcmd.2009.08.004 PMC3355376

[8] Zimran A (2011) How I treat Gaucher disease. Blood 118: 1463–71. 21670466 10.1182/blood-2011-04-308890

[9] Bodamer OA, Hung C (2010) Laboratory and genetic evaluation of Gaucher disease. Wien Med Wochenschr 160: 600–4. 20714811 10.1007/s10354-010-0814-1

[10] Vedder AC, Cox-Brinkman J, Hollak CE, Linthorst GE, Groener JE, et al (2006) Plasma chitotriosidase in male Fabry patients: a marker for monitoring lipid-laden macrophages and their correction by enzyme replacement therapy. Mol Genet Metab 89: 239–44. 16765076 10.1016/j.ymgme.2006.04.013

[11] Orchard PJ, Lund T, Miller W, Rothman SM, Raymond G, et al (2011) Chitotriosidase as a biomarker of cerebral adrenoleukodystrophy. J Neuroinflammation 8: 144. 22014002 10.1186/1742-2094-8-144 PMC3236018

[12] Hollak CE, van Weely S, van Oers MH, Aerts JM (1994) Marked elevation of plasma chitotriosidase activity. A novel hallmark of Gaucher disease. J Clin Invest 93: 1288–92. 8132768 10.1172/JCI117084 PMC294082

[13] Boot RG, Renkema GH, Verhoek M, Strijland A, Bliek J, et al (1998) The human chitotriosidase gene. Nature of inherited enzyme deficiency. J Biol Chem 273: 25680–5. 9748235 10.1074/jbc.273.40.25680

[14] Chang KL, Hwu WL, Yeh HY, Lee NC, Chien YH (2010) CCL18 as an alternative marker in Gaucher and Niemann-Pick disease with chitotriosidase deficiency. Blood Cells Mol Dis 44: 38–40. 19819171 10.1016/j.bcmd.2009.09.005

[15] Maire I, Guffon N, Froissart R (2007) Current development and usefulness of biomarkers for Gaucher disease follow up. Rev Med Interne 28: S187–92. 18228687 10.1016/s0248-8663(07)78880-x

[16] Schutyser E, Richmond A, Van Damme J (2005) Involvement of CC chemokine ligand 18 (CCL18) in normal and pathological processes. J Leukoc Biol 78: 14–26. 15784687 10.1189/jlb.1204712 PMC2665283

[17] Schutyser E, Struyf S, Proost P, Opdenakker G, Laureys G, et al (2002) Identification of biologically active chemokine isoforms from ascitic fluid and elevated levels of CCL18/pulmonary and activation-regulated chemokine in ovarian carcinoma. J Biol Chem 277: 24584–93. 11978786 10.1074/jbc.M112275200

[18] Struyf S, Schutyser E, Gouwy M, Gijsbers K, Proost P, et al (2003) PARC/CCL18 is a plasma CC chemokine with increased levels in childhood acute lymphoblastic leukemia. Am J Pathol 163: 2065–75. 14578205 10.1016/S0002-9440(10)63564-X PMC1892433

[19] Groener JE, Poorthuis BJ, Kuiper S, Hollak CE, Aerts JM (2008) Plasma glucosylceramide and ceramide in type 1 Gaucher disease patients: correlations with disease severity and response to therapeutic intervention. Biochim Biophys Acta 1781: 72–8. 18155675 10.1016/j.bbalip.2007.11.004

[20] Groener JE, Poorthuis BJ, Kuiper S, Helmond MT, Hollak CE, et al (2007) HPLC for simultaneous quantification of total ceramide, glucosylceramide, and ceramide trihexoside concentrations in plasma. Clin Chem 53: 742–7. 17332150 10.1373/clinchem.2006.079012

[21] Nilsson O, Svennerholm L (1982) Accumulation of glucosylceramide and glucosylsphingosine (psychosine) in cerebrum and cerebellum in infantile and juvenile Gaucher disease. J Neurochem 39: 709–18. 7097276 10.1111/j.1471-4159.1982.tb07950.x

[22] Dekker N, van Dussen L, Hollak CE, Overkleeft H, Scheij S, et al (2011) Elevated plasma glucosylsphingosine in Gaucher disease: relation to phenotype, storage cell markers, and therapeutic response. Blood 118: e118–27. 21868580 10.1182/blood-2011-05-352971 PMC3685900

[23] van Dussen L, Lips P, Everts VE, Bravenboer N, Jansen ID, et al (2011) Markers of bone turnover in Gaucher disease: modeling the evolution of bone disease. J Clin Endocrinol Metab 96: 2194–205. 21543432 10.1210/jc.2011-0162

[24] Rosengren B, Fredman P, Månsson JE, Svennerholm L (1989) Lysosulfatide (galactosylsphingosine-3-O-sulfate) from metachromatic leukodystrophy and normal human brain. J Neurochem 52: 1035–41. 2926387 10.1111/j.1471-4159.1989.tb01844.x

[25] Lloyd-Evans E, Pelled D, Riebeling C, Futerman AH (2003) Lyso-glycosphingolipids mobilize calcium from brain microsomes via multiple mechanisms. Biochem J 375: 561–5. 12917012 10.1042/BJ20030613 PMC1223730

[26] Atsumi S, Nosaka C, Iinuma H, Umezawa K (1993) Accumulation of tissue glucosylsphingosine in Gaucher-like mouse induced by the glucosylceramidase inhibitor cyclophellitol. Arch Biochem Biophys 304: 302–4. 8323295 10.1006/abbi.1993.1353

[27] Sun Y, Liou B, Ran H, Skelton MR, Williams MT, et al (2010) Neuronopathic Gaucher disease in the mouse: viable combined selective saposin C deficiency and mutant glucocerebrosidase (V394L) mice with glucosylsphingosine and glucosylceramide accumulation and progressive neurological deficits. Hum Mol Genet 19: 1088–97. 20047948 10.1093/hmg/ddp580 PMC2830832

[28] Auray-Blais C, Ntwari A, Clarke JT, Warnock DG, Oliveira JP, et al (2010) How well does urinary lyso-Gb3 function as a biomarker in Fabry disease? Clin Chim Acta 411: 1906–14. 20716442 10.1016/j.cca.2010.07.038

[29] Mao XY, Burgunder JM, Zhang ZJ, An XK, Zhang JH, et al (2010) Association between GBA L444P mutation and sporadic Parkinson’s disease from Mainland China. Neurosci Lett 469: 256–9. 20004703 10.1016/j.neulet.2009.12.007

[30] Taketomi T, Hara A, Uemura K, Sugiyama E (1996) Rapid method of preparation of lysoglycosphingolipids and their confirmation by delayed extraction matrix-assisted laser desorption ionization time-of-flight mass spectrometry. J Biochem 120: 573–9. 8902623 10.1093/oxfordjournals.jbchem.a021452

[31] Kurt I, Abasli D, Cihan M, Serdar MA, Olgun A, et al (2007) Chitotriosidase levels in healthy elderly subjects. Ann N Y Acad Sci 1100: 185–8. 17460177 10.1196/annals.1395.017

[32] Seeman PJ, Finckh U, Höppner J, Lakner V, Liebisch I, et al (1996) Two new missense mutations in a non-Jewish Caucasian family with type 3 Gaucher disease. Neurology 46: 1102–7. 8780099 10.1212/wnl.46.4.1102

[33] Finckh U, Seeman P, von Widdern OC, Rolfs A (1998) Simple PCR amplification of the entire glucocerebrosidase gene (GBA) coding region for diagnostic sequence analysis. DNA Seq 8: 349–56. 10728820 10.3109/10425179809020896

[34] Metz CE (1978) Basic principles of ROC analysis. Semin Nucl Med 8: 283–98. 112681 10.1016/s0001-2998(78)80014-2

[35] Zweig MH, Campbell G (1993) Receiver-operating characteristic (ROC) plots: a fundamental evaluation tool in clinical medicine. Clin Chem 39: 561–77. 8472349

[36] Delong ER, Delong DM, Clarke-Pearson DL (1988) Comparing the areas under two or more correlated receiver operating characteristic curves: a nonparametric approach. Biometrics 44: 837–45. 3203132

