# Supplementary material for: Glucosylsphingosine Is a Highly Sensitive and Specific Biomarker for Primary Diagnostic and Follow-Up Monitoring in Gaucher Disease in a Non-Jewish, Caucasian Cohort of Gaucher Disease Patients
Source: PLoS One. 2013 Nov 20;8(11):e79732. doi: 10.1371/journal.pone.0079732 (PMC3835853; doi:10.1371/journal.pone.0079732)
Supplement: Table S1 — Genotypes for all included GD patients. GBA cDNA accession number NP_000148.2; Traditional amino acid residue numbering, which excludes the first 39 aminoacids of the leader sequence, is provided and designed without the prefix “p”. All detected genotypes are listed and have been confirmed by Sanger sequencing. The cohort is comprised of a non-Jewish, Caucasian cohort of GD patients. (PDF) [file pone.0079732.s001.pdf]

Patient ID	cDNA Change	Aminoacid change	cDNA Change	Aminoacid change
4	c.860G>T	C287F	c.1226A>G	N370S
7	c.1226A>G	N370S	c.1448T>C	L444P
8	c.1226A>G	N370S	c.1448T>C	L444P
9	c.1448T>C	L444P		RecNciI
10	c.971G>A	R285H	c.1226A>G	N370S
14	c.362G>C	G82A	c.1226A>G	N370S
21	c.1226A>G	N370S		RecNciI
22	c.1226A>G	N370S		RecNciI
25	c.203delC	P29X	c.1226A>G	N370S
29	c.1226A>G	N370S		RecNciI
34		RecAP2		RecAP2
39	c.1448T>C	L444P	c.1483G>C	A456P
40	c.1226A>G	N370S		RecNciI
41	c.85dupG	A46term	c.1226A>G	N370S
43	c.155C>T	S13L	c.649C>T	P178S
45	c.1003C>G	L335V	c.1003C>G	L335V
47	c.1226A>G	N370S	c.1589C>T	T491I
48	c.1309G>C	V398L	c.1589C>T	T491I
49	c.1226A>G	N370S	IVS2(+1)	splice-site mutation
51	c.1448T>C	L444P	c.1448T>C	L444P
61	c.1226A>G	N370S	c.1309G>C	V398L
63	c.1448T>C	L444P	c.1504C>T	R502C
89	c.1448T>C	L444P	c.1448T>C	L444P
92	c.1226A>G	N370S	c.1309G>C	V398L
93	c.1448T>C	L444P	c.1483G>C	A456P
98	c.721G>A	G202R	c.886C>T	R257ter
101	c.1226A>G	N370S	c.1448T>C	L444P
102	c.721G>A	G202R	c.1226A>G	N370S
105	c.1226A>G	N370S	c.1342G>C	D409H
109	c.1448T>C	L444P		RecNciI
113	c.1226A>G	N370S	c.1505G>A	R502H
118	c.475C>T	R120W	c.1226A>G	N370S
123	c.1093G>A	Q326K		RecNciI + 1453G>C
124	c.1448T>C	L444P	c.1448T>C	L444P
125	c.677C>T	T226I	c.677C>T	T226I
126	c.1342G>C	D409H		RecNciI
137	c.1448T>C	L444P	c.1448T>C	L444P
139	c.1226A>G	N370S	c.1448T>C	L444P
142	c.1226A>G	N370S	c.1448T>C	L444P
145	c.1226A>G	N370S	c.1226A>G	N370S
149	c.721G>A	G202R	c.1226A>G	N370S
154	c.437C>T	S146L	c.1226A>G	N370S
159	c.882T>G	H255Q	c.1226A>G	N370S
162	c.1226A>G	N370S	c.1448T>G	L444R
163	c.1226A>G	N370S	c.1448T>G	L444R
165	c.394A>T	I93F	c.1193G>A	R359Q
168	c.1448T>C	L444P	c.1448T>C	L444P
170	c.1448T>C	L444P	c.1448T>C	L444P
176	c.827C>T	S237F	c.1448T>C	L444P
178	c.809C>G	T231R	c.1226A>G	N370S
181	c.1226A>G	N370S	c.1448T>C	L444P
184	c.1088T>C	L324Q	c.1226A>G	N370S

Patient ID	cDNA Change	Aminoacid change	cDNA Change	Aminoacid change
187	c.476G>A	R120Q	c.1226A>G	N370S
193	c.482C>G	P161R	c.1226A>G	N370S
196	c.1448T>C	L444P	c.1448T>C	L444P
198	c.1226A>G	N370S	c.1226A>G	N370S
203	c.1181G>C	G355A	c.1181G>C	G355A
206	c.379G>C	A88P	c.1226A>G	N370S
209	c.667T>C	W184R	c.1226A>G	N370S
211	c.1226A>G	N370S		RecNciI
212	c.1226A>G	N370S		RecNciI
229	c.1226A>G	N370S	c.203_204insC	P68fs
230	c.1448T>C	L444P	c.1448T>C	L444P
235	IVS3+1G>A	splice-site mutation	c.1223C>T	T369M
236	c.1342G>C	D409H	c.1448T>C	L444P
237	c.1448T>C	L444P		RecNciI
239	c.1226A>G	N370S	c.818A>G	N234S
240	c.1448T>C	L444P	c.1448T>C	L444P
241	c.1226A>G	N370S	c.1226A>G	N370S
243	c.1226A>G	N370S	c.1448T>C	L444P
244	c.971G>A	R285H	c.1226A>G	N370S
245	c.1208G>A	S403N	c.1226A>G	N370S
249	c.1226A>G	N370S	c.1448T>C	L444P
250	c.1448T>C	L444P	IVS2(+1)	splicing
251	c.1093G>A	Q326K	c.1226A>G	N370S
252	c.764T>A	F216Y	RecTL-c1263del	
253	c.476G>A	R120Q	c.1226A>G	N370S
254	c.1226A>G	N370S	c.1448T>C	L444P
255	c.1226A>G	N370S		RecNciI
256	c.1226A>G	N370S	c.1226A>G	N370S
257	c.1226A>G	N370S		RecNciI
258	c.809C>G	T231R	c.1226A>G	N370S
259		RecAH3		RecNciI
342	c.1240G>T	V375L	c.1448T>C	L444P
381	c.1448T>C	L444P	c.1448T>C	L444P
844	c.1453G>C	A485P	c.1453G>C	A446P
917	c.1226A>G	N370S	c.1448T>C	L444P
918	c.115+1G>A	splice-site mutation	c.1226A>G	N370S
919	c.115+1G>A	splice-site mutation	c.1226A>G	N370S
920	c.1226A>G	N370S	c.1226A>G	N370S
921	c.1448T>C	L444P	c.1448T>C	L444P
922	c.115+1G>A	splice-site mutation	c.1226A>G	N370S
923	c.754T>A	F213I	c.1226A>G	N370S
924	c.1226A>G	N370S	c.1448T>C	L444P
925	c.1184C>T	S356F	c.1448T>C	L444P
927	c.115+1G>A	splice-site mutation	c.1226A>G	N370S
928	c.1226A>G	N370S	c.1448T>C	L444P
929	c.1226A>G	N370S	c.1448G>C	L444P
938	c.1448T>C	L444P	c.1448T>C	L444P
942	c.1448T>C	L444P	c.1448T>C	L444P
988		RecFK		RecNciI
989	c.1226A>G	N370S	c.1448T>C	L444P
990	c.1226A>G	N370S	c.1448T>C	L444P
992	c.85dupG	L29Afs*18	c.1226A>G	N370S

Patient ID	cDNA Change	Aminoacid change	cDNA Change	Aminoacid change
993	c.1226A>G	N370S	c.1226A>G	N370S
1028	c.930T>A	S271R	c.1448T>C	L444P
1034	c.1226A>G	N370S	c.1226A>G	N370S
1035	c.494-1G>A	C126Y	c.847T>C	Y244H
1037	c.1226A>G	N370S	c.1448T>C	L444P
1044	c.1448T>C	L444P	c.1448T>C	L444P
1046	c.1226A>G	N370S	c.1448T>C	L444P
1047	c.1226A>G	N370S	c.1448T>C	L444P
1048	c.115+1G>A	splice-site mutation	c.1226A>G	N370S
1049	c.1226A>G	N370S	c.1448T>C	L444P
1050	c.754T>A	F213I	c.1226A>G	N370S
1051	c.85dupG	L29Afs*18	c.1226A>G	N370S
1052	c.1226A>G	N370S	c.1226A>G	N370S
1053	c.115+1G>A	splice-site mutation	c.1226A>G	N370S
1054	c.85dup	L29Afs*18	c.1226A>G	N370S
1055	c.1448T>C	L444P	c.1448T>C	L444P
1056	c.1448T>C	L444P	c.1448T>C	L444P
1057	c.115+1G>A	splice-site mutation	c.1226A>G	N370S
1064	c.509G>T	R131L	c.1226A>G	N370S
1066	c.1448T>C	L444P	c.1448T>C	L444P
1068	c.754T>A	F213I	c.1365G>A	M416I
1069	c.754T>A	F213I	c.1365G>A	M416I
1070	c.754T>A	F213I	c.1365G>A	M416I
1071	c.754T>A	F213I	c.1365G>A	M416I
